# Between Likes and Ethics: Benefits and Challenges of Social Media Use in Medical Practice in a Pre- and Post-intervention Study

**DOI:** 10.7759/cureus.116279

**Published:** 2026-09-15

**Authors:** Pedro Enzo Camargo Luz, Luiza Letti Ferronatto, Pedro Paulo Caixeta

**Affiliations:** 1 Medicine, Centro Universitário de Brasília, Brasília, BRA

**Keywords:** cfm resolution, digital professionalism, medical education, medical ethics, social media

## Abstract

Background: Social media use among medical students is nearly universal, yet it often occurs without adequate ethical-legal literacy, particularly considering the update introduced by Resolution No. 2,336/2023 of the Brazilian Federal Council of Medicine (Conselho Federal de Medicina, CFM). This study assessed medical students’ knowledge and perceptions of the benefits, risks, and ethical limits of social media use before and after an educational intervention based on practical scenarios grounded in that resolution.

Methodology: This was a quasi-experimental study with a paired pre- and post-test design. An electronic questionnaire containing 15 practical scenarios rated on a five-point Likert scale (13 forming the ethical adequacy score and two assessing institutional/curricular perceptions) was administered to students from the first through the twelfth semester. After excluding colliding pairing codes, 101 valid matched pairs were analyzed. Unethical responses were reverse-scored to build an ethical adequacy score, compared using the Wilcoxon signed-rank test (primary analysis, given a deviation from normality) and a paired Student’s t-test (sensitivity analysis).

Results: The overall ethical adequacy score rose from a mean of 3.40 (standard deviation (SD) 0.40) to 3.95 (SD 0.42) after the workshop (*P* < 0.001, Cohen's *d* = 1.37, 95% confidence interval (CI) 0.47-0.63), with the largest gains observed in scenarios addressing therapeutic guidance via direct message, advertising sensationalism, and exposure of surgical procedures. Two items describing already-permitted conduct (regulatory flexibilities and the use of social media as a communication channel during an epidemic) showed a significant score decrease, indicating a post-intervention overconservatism effect. No significant differences in score change were found between subgroups defined by training stage, gender, or daily screen time.

Conclusions: Scenario-based interventions are effective in raising ethical adequacy in the short term, but they require monitoring for interpretive side effects and should be complemented by continuous curricular training.

## Introduction

Over the past few decades, digital transformation has profoundly changed how people communicate, generate information, and build social relationships. In healthcare, these changes have gone beyond the incorporation of new clinical technologies to influence communication among physicians, patients, and society at large. Social media are no longer spaces reserved for personal interaction; they have become part of medical practice itself, used for health education, scientific dissemination, professional branding, and closer engagement with the public. Platforms such as Instagram, TikTok, and YouTube have expanded the reach of health information and created new possibilities for communication between health professionals and the population.

This scenario, however, has introduced ethical challenges that were far less pronounced before these platforms became widespread. The ease of producing and sharing content has increased the risk of violations involving professional confidentiality, patient privacy, medical advertising, and the commercialization of the profession. Content posted on social media can also generate lasting legal and ethical consequences, since it remains permanently recorded and can reach a large audience within minutes. As a result, the practice of medicine now requires competencies that go beyond technical-scientific training, including knowledge of the rules governing professional conduct in the digital environment.

Digital transformation has irreversibly permeated both medical education and clinical practice, establishing digital platforms not only as socialization tools but also as resources for teaching, research, health promotion, and patient engagement. Reported usage rates among medical students exceed 90% [1-3]. Within this hyperconnected landscape, the concept of digital professionalism (e-professionalism) has emerged, describing how the traditional ethical values of the medical profession are adapted to the virtual environment, requiring future physicians to maintain probity, confidentiality, and integrity in their digital interactions [1-3].

Medical students, as digital natives, report substantial screen time, routinely spending one to six hours per day on platforms such as Instagram, WhatsApp, YouTube, TikTok, and X (formerly Twitter). These tools are used in a hybrid manner, combining purely personal purposes with educational and professional goals such as scientific updating and early digital authority-building. Students broadly recognize that social media play a key role in healthcare today and that informative posts have real potential to encourage healthier habits among patients [4-6].

Despite this technological familiarity and theoretical recognition of the topic’s importance, a marked paradox emerges in medical training: high connectivity is frequently accompanied by low digital ethical literacy. Studies identify a clear cognitive dissonance among undergraduates: although most agree that online professionalism is as important as offline professionalism and that inappropriate conduct affects professional reputation, many paradoxically believe that their social media activity will not affect their careers. A significant proportion of students also defend unrestricted freedom of action online, rejecting the idea that academic institutions or medical councils have the right to intervene in or regulate their personal profiles [6,7].

This gap between ethical theory and virtual practice is worsened by the so-called *online disinhibition effect*. A false sense of privacy, together with the apparent absence of immediate consequences in the digital environment, encourages unprofessional behaviors that students would never adopt during in-person clinical care. This erosion of professionalism is reinforced by the *hidden curriculum* encountered during clerkships and internships: observing preceptors and resident physicians engage in inappropriate conduct, such as searching for patients on search engines without clinical justification or using unencrypted messaging applications, leads students to normalize and reproduce such practices, often outweighing the effect of isolated theoretical lectures on the topic [8,9].

Ethical perception and conduct also fluctuate with academic maturation. Pre-clinical students tend to be more permissive regarding exposure to hospital environments and digital interaction with patients, driven by early enthusiasm. As students advance into the clinical cycle and internship, closer contact with patients and real dilemmas should, in principle, induce greater caution. Paradoxically, the familiarity gained during internship is frequently associated with an increased incidence of borderline conduct, such as accepting friend requests from patients or discussing clinical cases in personal messaging groups, suggesting that practical experience, without adequate transversal guidance, can weaken the rigor of professional boundaries [10,11].

Consequently, the blurring of boundaries between personal life and professional medical identity online results in inappropriate exposure, breaches of confidentiality, and posts that compromise the credibility of the profession. The literature suggests adopting a model of digital *dual citizenship*, based on strict separation between personal and professional profiles, together with strategies focused on maintaining appropriate distance and minimizing risk. For e-professionalism to become firmly established, however, the formal implementation of digital health literacy programs within curricula is essential, equipping students to translate traditional ethical norms into the reality of their screens [3,4,12].

Professional confidentiality is one of the pillars of medical ethics, a legal and moral duty that has underpinned trust in the therapeutic relationship since the Hippocratic tradition. The Brazilian Code of Medical Ethics expressly forbids disclosing facts learned in the course of professional practice, except for just cause, legal obligation, or written consent. The ease of sharing content afforded by social media, however, has exposed patients to new risks of confidentiality breaches. Publishing photographs, videos, test results, and clinical case discussions on open platforms or informal groups frequently occurs without adequate de-identification, constituting a serious ethical violation. Patient consent alone does not authorize sensationalist exposure or the display of images that make the patient identifiable in an inappropriate way; strict limits apply to the disclosure of sensitive data, even under the justification of educational purposes [13,14].

Another contemporary challenge lies in the fluid boundaries of the physician-patient relationship in the virtual environment, particularly through instant messaging applications such as WhatsApp and direct-message services. While these tools provide agility in communication and support in emergencies, the literature points to risks of trivializing medical care, invading the professional’s privacy, and eroding boundaries set by users. Informal tele-guidance and prescriptions issued through unofficial channels, unaccompanied by a complete medical act - requiring history-taking, physical or virtual assessment, and secure medical-record documentation - compromise the legal and ethical safety of care. Telemedicine regulations require that remote care follow strict protocols for security, explicit consent, and registration in an Electronic Health Record System, conditions frequently neglected in informal digital communication [15,16].

In advertising, Brazilian medicine has historically adopted restrictive positions to curb the commercialization of healthcare, unfair competition, and improper patient solicitation. The Code of Medical Ethics prohibits sensationalism, excessive self-promotion, and, above all, the promise or guarantee of results. Attributing privileged capability to particular methods or devices is strictly forbidden, protecting patients from unrealistic expectations and misleading advertising. Managing one’s digital identity is also an ongoing challenge; the absence of separation between personal and professional profiles, combined with posts involving alcohol consumption, parties, or decontextualized humor, can damage the image of the profession and the physician’s credibility before society [4,13].

In response to this transformation, the Brazilian Federal Council of Medicine updated its guidelines through Resolution No. 2,336/2023, establishing a new regulatory framework for medical advertising. The resolution introduced significant flexibilities, permitting *before-and-after* images of procedures and the re-posting of patient testimonials, provided the content remains strictly educational, is presented soberly, and preserves patient anonymity (even when a prior consent form has been signed). The resolution also authorized the disclosure of consultation fees and promotional campaigns, provided these are not tied to bundled sales, and regulated the use of stock image banks with due copyright citation [17].

Despite these concessions, the new regulatory landscape maintained and reinforced essential prohibitions. Guaranteeing results and publishing manipulated or edited clinical images remain strictly forbidden. Clear identification of the professional is now mandatory in all advertising material, including the physician’s name, the term *physician*, the medical license number, and, when applicable, board-certified specialty status; physicians holding a non-specialist postgraduate degree may advertise related content only if they clearly state *not a board-certified specialist*. Capturing and publishing images of surgical procedures by external film crews remains authorized only in very specific situations (such as childbirth, with the patient’s consent), reaffirming that teaching of exclusive medical techniques must remain restricted to academic and professional settings, away from platforms open to a lay audience [17].

Despite these regulatory innovations, terms such as *sobriety* in testimonials and *sensationalism* in advertising carry considerable subjectivity, generating ambiguous, gray-area situations between legitimate scientific dissemination and self-promotion. This difficulty in applying newly established rules objectively shows that digital ethical literacy is not an intuitive competency, even for generations of students immersed in technology from an early age, reinforcing the need for specific training, case discussions, and continuous updating within medical schools [17].

The teaching of ethics in medical schools has historically focused on the classic principles of bioethics and medical law, oriented toward in-person clinical practice. Curricula, however, frequently fail to keep pace with technological change, leaving a considerable gap in digital ethical literacy. Most students report never having received specific classes or tutorials on online professionalism during their training. As a result, although digital natives, students operate in a zone of legal and ethical insecurity, blurring the boundaries between the personal and professional use of social media [6,9,18].

Merely reading codes of ethics or offering isolated single-semester theoretical courses has proven insufficient to ensure the expected behavioral adequacy of future physicians. A deep assimilation of professionalism depends on a transversal, continuous, and practical approach throughout the course, particularly during internship. The absence of a solid curricular axis and clear institutional guidelines leaves students vulnerable to online disinhibition and to reproducing the unethical conduct observed through the hidden curriculum [7,9,14].

To close this gap, educational interventions based on active learning methodologies - including case-based discussion, scenario simulation, and reflective sessions - have proven highly effective, producing statistically significant gains in ethical discernment and decision-making when assessed with pre- and post-test designs. Such short, practical workshops outperform traditional lectures because they allow students to operationalize abstract professionalism concepts and correct misconceptions in real time [9,19]. Despite this international evidence, quantitative Brazilian studies using a pre-/post-test design to evaluate the immediate impact of interventions focused specifically on digital ethics and medical-advertising regulations remain scarce [18,19], which is the gap this study aims to address.

The objectives of this study were to: (1) evaluate the baseline knowledge of medical students regarding digital ethics and professional boundaries; (2) assess the short-term impact of a brief educational intervention on their ethical adequacy scores using hypothetical scenarios; and (3) identify specific areas of digital professionalism that require further educational focus.

## Materials and methods

This was a quasi-experimental intervention study with a paired pre- and post-test design and a quantitative approach, designed to evaluate the impact of a brief educational intervention. The study population consisted of students regularly enrolled in the medical program at Centro Universitário de Brasília (CEUB), from the first through the twelfth semester. Inclusion criteria were agreement with the informed consent form and completion of both the pre- and post-intervention questionnaires, which were made available in person via QR code and link during the intervention (Appendices A-B). Exclusion criteria were withdrawal after enrollment, incomplete completion of either questionnaire, and impossibility of matching the self-generated alphanumeric identification codes.

Data collection initially yielded 130 pre-class and 117 post-class responses. After applying exclusion criteria (mainly related to student attrition before the second stage or code-entry discrepancies), an initial matched sample of 108 participants was obtained. A further code review identified that seven of these matched pairs actually corresponded to code collisions between different students who coincidentally generated the same alphanumeric code (same first two letters of the mother’s name and birth date), with diverging semester and/or gender, rather than resubmissions by the same student. Because this corresponds precisely to the *impossibility of matching* the exclusion criterion defined in the protocol, these seven codes were excluded from both sides of the dataset, resulting in a final valid matched sample of 101 participants (*n* = 101).

Data were collected in person using structured questionnaires built and administered through Google Forms. The methodological workflow comprised three sequential stages. First, a pre-class questionnaire collected the sociodemographic profile and assessed baseline ethical knowledge through 13 practical scenarios rated on a five-point Likert scale (from *strongly disagree* to *strongly agree*), plus two additional items on subjective perceptions of the students’ own training that were not included in the ethical adequacy score. Next, a lecture addressed the main digital dilemmas and the regulatory updates introduced by Resolution No. 2,336/2023; the lecture did not provide direct answers to the scenarios in the questionnaire, and it used different cases and examples so that students had to apply the ethical reasoning just acquired rather than recall memorized answers. The educational intervention consisted of a standardized workshop lasting approximately 40 minutes in total, which included a 25-minute lecture and 15 minutes allocated for the completion of the pre- and post-intervention questionnaires. The content covered core medical ethics principles applied to digital environments, utilizing multimedia presentations and interactive case-based discussions. Immediately after the intervention, the same scenarios were reapplied in a post-class questionnaire to reliably measure each participant’s immediate knowledge gain, judgment ability, and change in perspective. To allow longitudinal tracking of responses without compromising anonymity, each participant created a self-generated alphanumeric identifier composed of the first two letters of the participant’s mother’s name followed by the participant’s day of birth.

Collected data were tabulated and submitted to statistical analysis. Likert-scale response options were converted into ordinal numeric variables from 1 to 5. A reverse-scoring technique was applied to the ten scenarios describing unethical conduct or conduct prohibited by the Federal Council of Medicine, ensuring that higher numeric values invariably reflected greater ethical adequacy; the three scenarios describing already-ethical conduct retained their original direction, together forming a 13-item ethical adequacy score. This novel metric was developed by the authors specifically for this study to quantitatively assess the participants' alignment with the digital conduct guidelines established by CFM Resolution No. 2,336/2023. To ensure reproducibility, this scoring rubric was strictly standardized, assigning specific point values to responses that aligned with current medical council guidelines regarding patient privacy and professional boundaries.

The intervention’s impact was assessed using the Wilcoxon signed-rank test for paired data, evaluating the sample’s change in performance on each scenario. The paired Student’s t-test was used as a complementary sensitivity analysis; however, because the Shapiro-Wilk test indicated a deviation from normality in the post-intervention distribution (*P* = 0.012), the Wilcoxon test was adopted as the primary test for all comparisons. Differences were considered statistically significant at *P *< 0.05. To comply with rigorous reporting standards, 95% confidence intervals (CIs) and appropriate effect size measures (such as Cohen's *d* for t-tests and the rank-biserial correlation for Wilcoxon tests) were calculated for all comparisons, along with degrees of freedom where applicable. Database organization and statistical analyses were performed in the Python programming language, using the Pandas, NumPy, and SciPy libraries.

The study was conducted in accordance with the ethical and legal principles governing research involving human subjects in Brazil, following the guidelines of Resolution No. 466/2012 of the Brazilian National Health Council. The project was submitted to and approved by the CEUB Research Ethics Committee (Certificate of Presentation for Ethical Consideration (CAAE): 91753125.2.0000.0023; Opinion No. 7,875,799, dated October 1, 2025). All participants confirmed their awareness of and agreement with an electronic informed consent form, which was made available both within the questionnaire and via QR code at the time of the intervention, and completion of which was a mandatory first step before access to the survey questions was granted. Confidentiality of information and the right to withdraw at any time were guaranteed. Collected data were stored in a secure, two-factor-authenticated Google account created exclusively for the study and accessible only to the researchers, with scheduled permanent deletion three years after study completion. Participant identity was protected through questionnaire anonymization and pair-code encoding, actively mitigating risks of confidentiality breaches or data leakage.

Generative AI tools (Gemini and NotebookLM) were used to assist with translation of the abstract, language and grammar editing, and textual organization during preparation of the original Portuguese-language report. All statistical analyses described above were performed independently by the authors using Python, without AI assistance.

## Results

The final study sample comprised 101 medical students assessed in a paired manner before and after the educational intervention. The sociodemographic analysis showed a predominance of female participants (69, 68.3%) compared with male participants (32, 31.7%). Mean age was 22.9 years (SD 3.6; median 23; range 18-36). The sample spanned different stages of medical training: 34 (33.7%) participants were in the basic cycle (1st-4th semester), 30 (29.7%) in the clinical cycle (5th-8th semester), and 37 (36.6%) in internship (9th-12th semester), as shown in Table 1.

**Table 1:** Sociodemographic and academic profile of the students assessed (n = 101). *Birth-date records for two participants showed a data-entry inconsistency (birth year registered as 2026, incompatible with the collection date) and were treated as missing exclusively for the age calculation (*n* = 99 for this variable), with no impact on the remaining analyses. SD, standard deviation

Variable	n	%
Sex		
Female	69	68.3
Male	32	31.7
Training stage		
Basic cycle (1st-4th semester)	34	33.7
Clinical cycle (5th-8th semester)	30	29.7
Internship (9th-12th semester)	37	36.6
Age (years)*		
Mean (SD)	22.9 (3.6)	-
Median	23	-
Range (min-max)	18-36	-

Digital-presence habits showed that social media use is nearly universal among these future professionals: 94 (93.1%) reported having active profiles. Instagram was the dominant platform (95, 94.1%), followed by TikTok (68, 67.3%) and YouTube (61, 60.4%). Daily screen time was substantial: 51 (50.5%) of students reported 1-3 hours per day and 30 (29.7%) reported 3-5 hours per day - that is, 81 (80.2%) of the sample used social media for at least one and up to five hours daily. Despite this high connectivity, only 34 (33.7%) of the sample reported using social media specifically for medicine-related activities, such as health information dissemination or early professional marketing.

Baseline knowledge of ethical norms revealed a paradox central to this study. When asked whether they had heard of the Federal Council of Medicine (CFM) rules on social media use, 72 (71.3%) answered affirmatively; however, this superficial theoretical familiarity did not translate into technical mastery or practical confidence (Figure 1).

**Figure 1:**
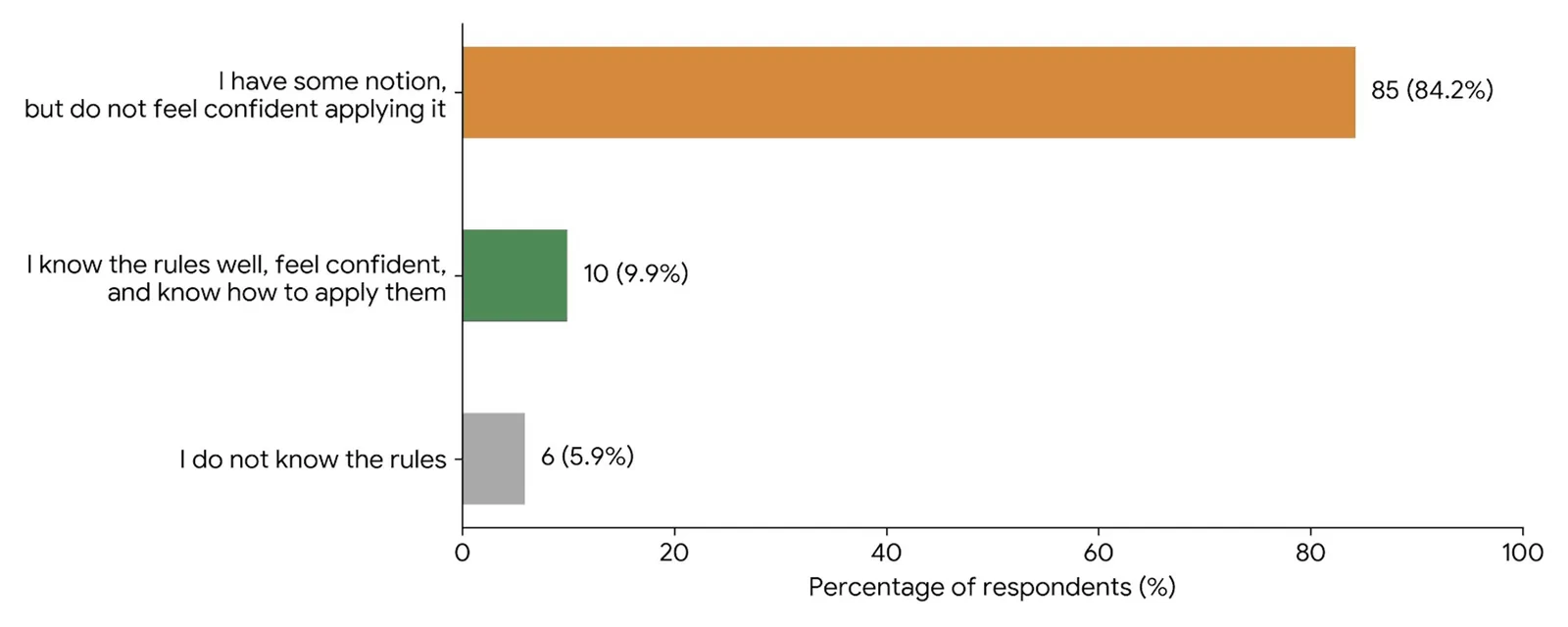
Self-perceived technical and ethical safety regarding CFM (Conselho Federal de Medicina, Brazilian Federal Council of Medicine) Resolution No. 2,336/2023 among medical students at baseline, before the educational intervention (n = 101).

Baseline self-perception showed that most students (85, 84.2%) reported having some notion of the rules but not feeling confident applying them in clinical practice. Only 10 (9.9%) reported knowing the rules well enough to apply them safely, and 6 (5.9%) reported not knowing them at all. This baseline scenario suggests that, although students are digital natives, the ethical-legal literacy required for professional practice does not naturally follow from technological familiarity.

To measure the objective impact of the workshop, Likert-scale responses (1-5) were submitted to the Wilcoxon signed-rank test, with the paired Student’s t-test as a sensitivity analysis. Reverse scoring was applied to the 10 scenarios describing unethical conduct or conduct prohibited by the CFM, while the original direction was kept for the three scenarios describing already-ethical conduct, together composing a 13-item ethical adequacy score in which higher scores (closer to 5) invariably reflected greater alignment with the medical council’s norms. The full pre-intervention response distribution is shown in Figure 2 and re-expressed as a diverging agree/disagree chart in Figure 3.

**Figure 2:**
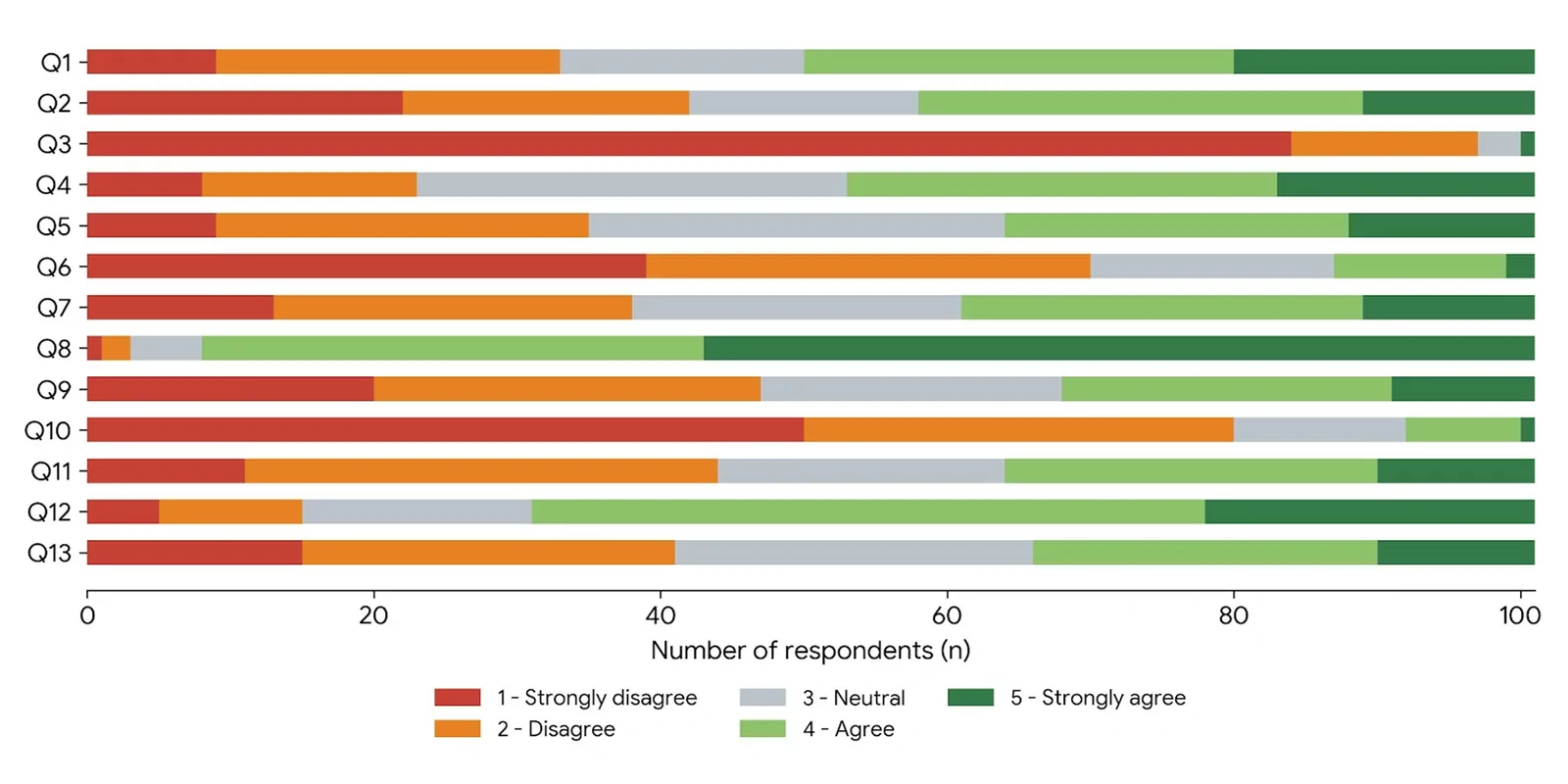
Distribution of pre-intervention responses (five-point Likert scale, 1-5) for the 13 scenarios composing the ethical adequacy score (n = 101).

**Figure 3:**
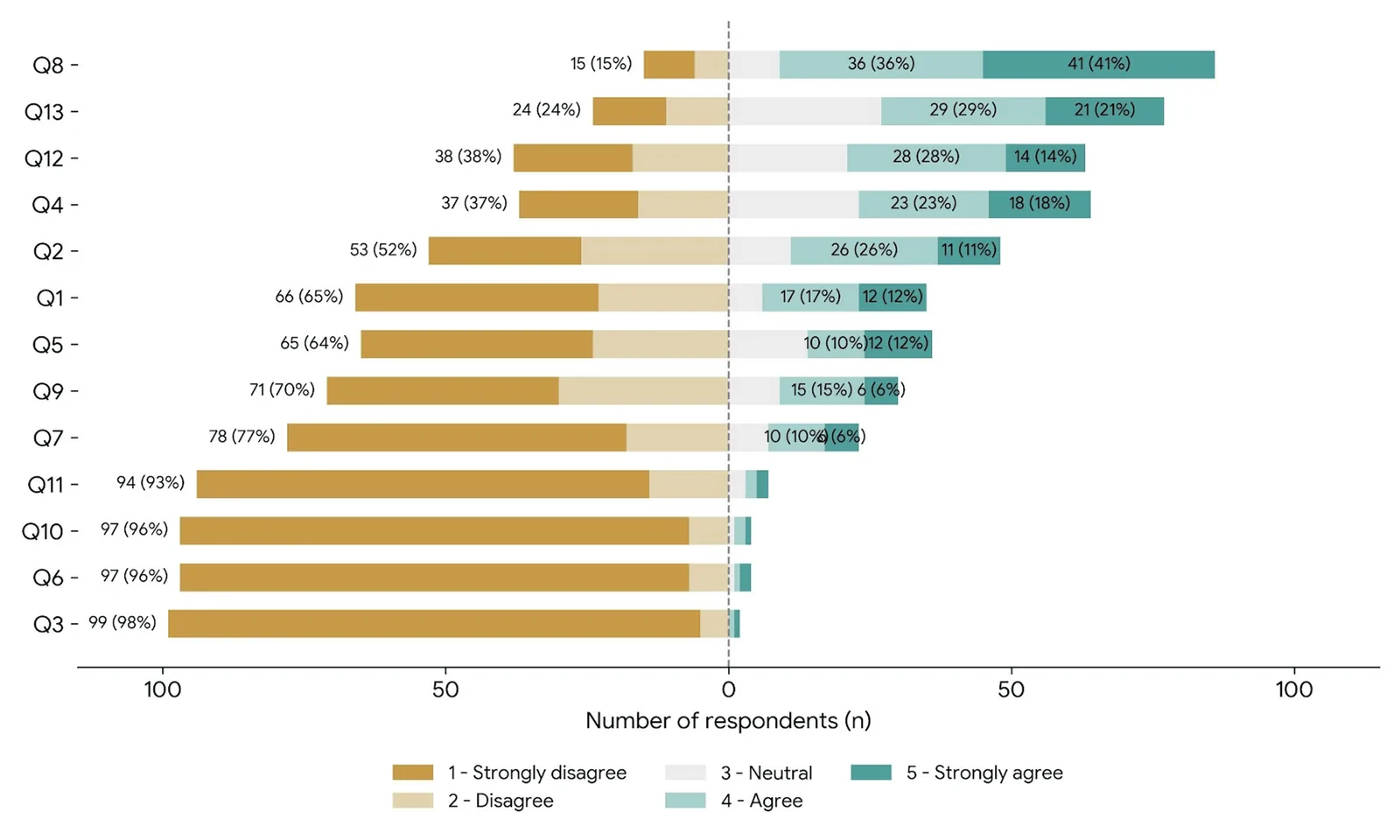
Pre-intervention responses to the 13 evaluated clinical scenarios, re-expressed as a diverging chart grouping responses into disagreement (categories 1-2), neutral (category 3), and agreement (categories 4-5), sorted by total agreement (n = 101).

Statistical analysis showed a significant knowledge gain. The sample’s overall ethical adequacy score rose from a mean of 3.40 (SD 0.40; median 3.38) to 3.95 (SD 0.42; median 4.08) post-class, a statistically significant difference (Wilcoxon, *P* < 0.001, rank-biserial correlation *r* = 0.88, 95% CI of the median difference (0.45-0.65); paired t-test, *t*(100) = 13.75, *P* < 0.001, Cohen's *d* = 1.37, 95% CI of the mean difference (0.47-0.63)) (Figure 4). The corresponding post-intervention item-level response distribution is shown in Figures 5-6.

**Figure 4:**
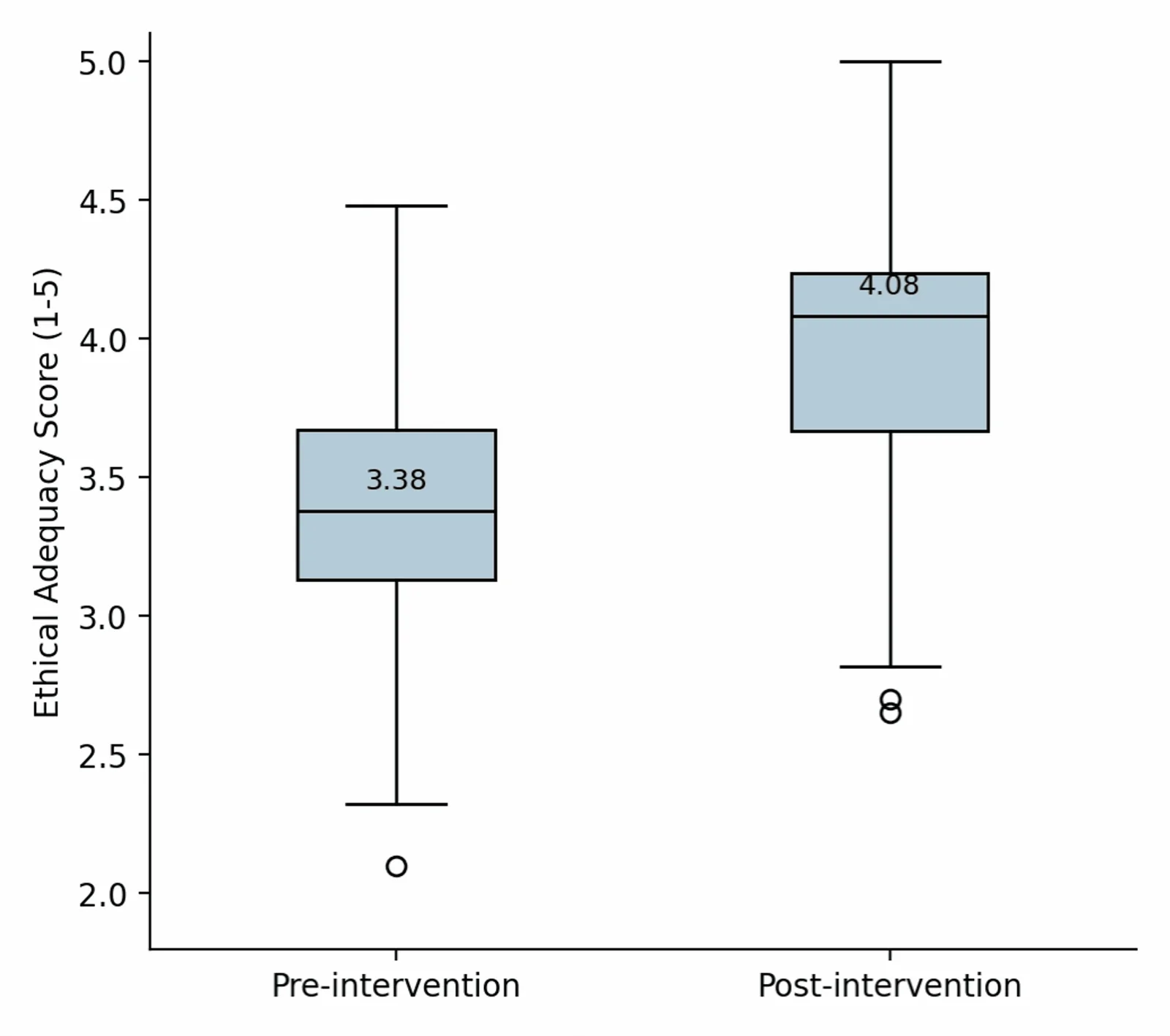
Ethical Adequacy Score distribution before and after the educational intervention (n = 101 paired). Boxes show the median and interquartile range (IQR); whiskers extend to 1.5× IQR; open circles denote values beyond the whiskers. Pre-intervention: mean 3.40 (SD, standard deviation, 0.40), median 3.38; post-intervention: mean 3.95 (SD 0.42), median 4.08 (Wilcoxon signed-rank test, *P* < 0.001; paired Student’s t-test, *t*(100) = 13.75, *P* < 0.001). Box edges and whiskers were reconstructed from the reported mean, SD, and median for each time point; individual paired data points from the original analysis are not reproduced in this version of the figure.

**Figure 5:**
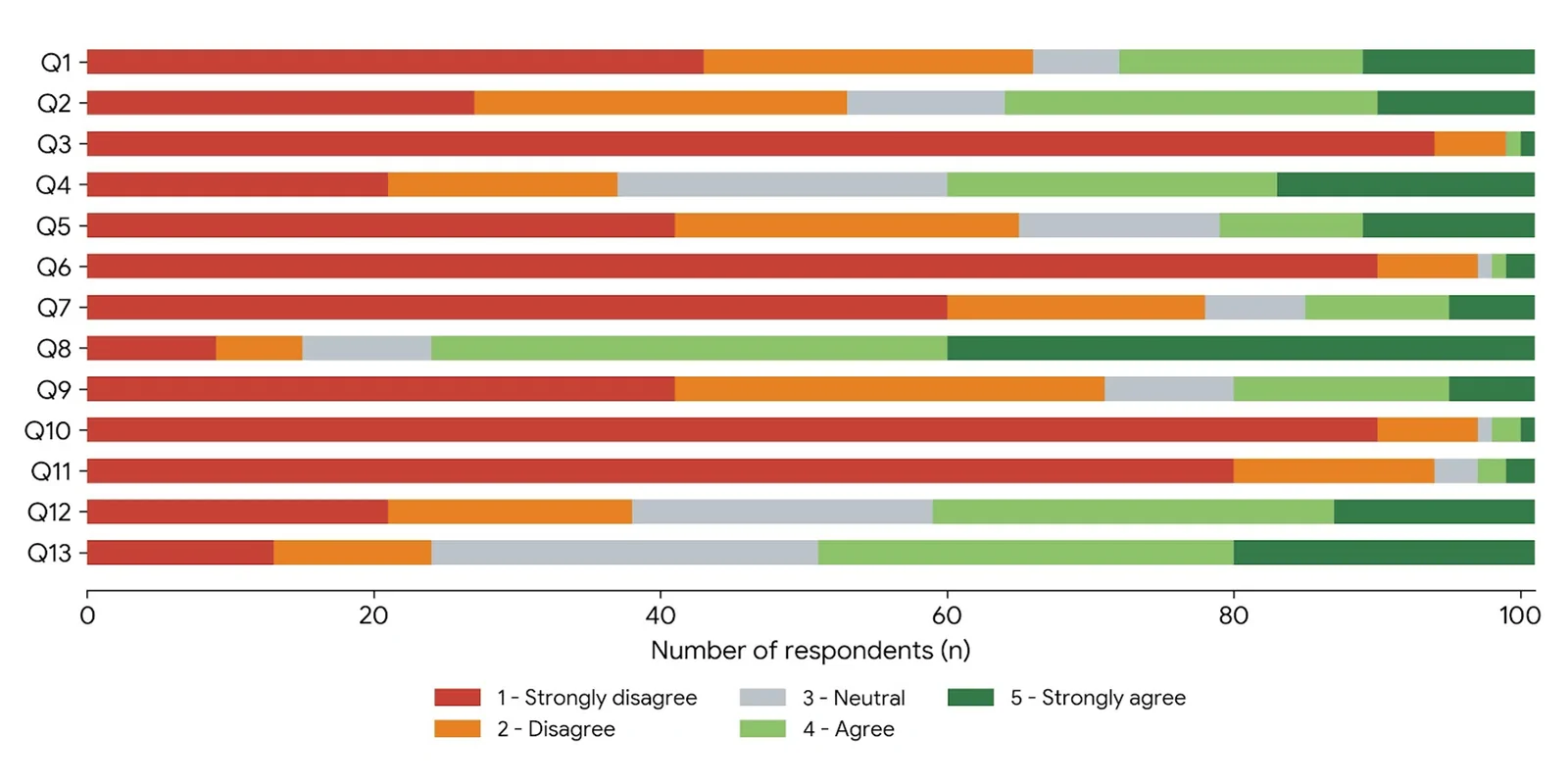
Distribution of post-intervention responses (five-point Likert scale, 1-5) for the 13 evaluated clinical scenarios (n = 101).

**Figure 6:**
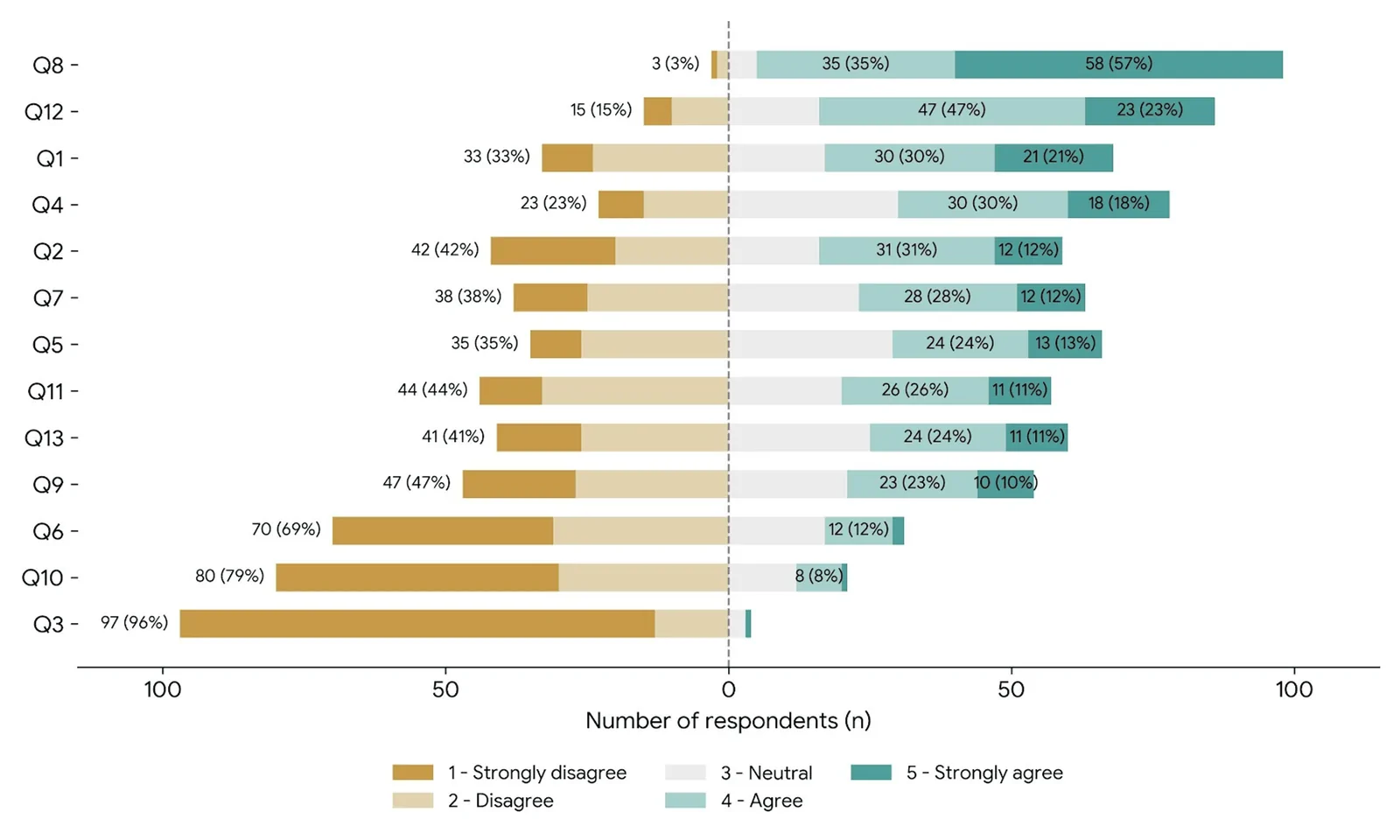
Post-intervention responses to the 13 evaluated clinical scenarios, re-expressed as a diverging chart grouping responses into disagreement (categories 1-2), neutral (category 3), and agreement (categories 4-5), sorted by total agreement (n = 101).

Item-level analysis showed the largest correction occurred in the scenario simulating therapeutic guidance via direct message (answering questions about medication side effects through Instagram direct messages), which rose from 3.12 to 4.67 (Wilcoxon, *P* < 0.001, *r* = 0.91, 95% CI 1.3-1.8). The item on advertising sensationalism (*only clinic in the city* equipped with a given device) also showed a significant increase, from 3.93 to 4.83 (*P* < 0.001, *r* = 0.85, 95% CI 0.7-1.1). Exposure to surgical procedures by students or residents on social media, even for teaching purposes, rose from 3.05 to 4.16 (*P* < 0.001, *r* = 0.82, 95% CI 0.9-1.3). For the absolute prohibition on guaranteeing results, the baseline score was already high (4.80), close to the scale’s ceiling, though the intervention still produced a statistically significant increase (4.80-4.92; *P* = 0.026, *r* = 0.45, 95% CI 0.05-0.25), as detailed in Table 2.

**Table 2:** Ethical adequacy score by item (n = 101, Wilcoxon signed-rank test).

Item	Scenario (summary)	Scoring	Pre	Post	P
Q1	Photograph of a wound posted with verbal consent	Reverse-scored	2.76	3.72	<0.001
Q2	Student group shares anonymized medical records	Reverse-scored	3.12	3.31	0.137
Q3	Guarantee of 0% error rate	Reverse-scored	4.80	4.92	0.026
Q4	Social media as a valid channel during an epidemic	Direct-scored	3.31	2.99	0.011
Q5	Profile used exclusively for medical content	Reverse-scored	2.98	3.75	<0.001
Q6	*Only clinic in the city* advertising claim	Reverse-scored	3.93	4.83	<0.001
Q7	Step-by-step surgical video	Reverse-scored	3.05	4.16	<0.001
Q8	Non-specialist advertising a non-titled subspecialty area	Direct-scored	4.43	3.96	<0.001
Q9	De-identified real clinical case from an on-call shift	Reverse-scored	3.28	3.85	<0.001
Q10	Phrases such as *best treatment*	Reverse-scored	4.22	4.82	<0.001
Q11	Therapeutic guidance via direct message	Reverse-scored	3.12	4.67	<0.001
Q12	Photo of an empty operating room	Reverse-scored	2.32	3.01	<0.001
Q13	Medical humor content harming professional image	Direct-scored	2.90	3.36	<0.001

Two items followed a paradoxical pattern of decrease. The scenario addressing recent regulatory flexibilities - a non-specialist physician advertising treatment of certain conditions, provided the absence of board certification is clearly stated - fell from 4.43 to 3.96 (*P* < 0.001, *r* = -0.65, 95% CI -0.7 to -0.2). The item on social media as a valid channel for transmitting reliable medical information during an epidemic also fell significantly, from 3.31 to 2.99 (*P* = 0.011, *r* = -0.48, 95% CI -0.5 to -0.1), despite describing a legitimate and already-recognized use of digital health communication.

One scenario showed no statistically significant change: the possibility of a student group sharing de-identified clinical cases, including test and record images, on a social media page for learning purposes (3.12-3.31; *P* = 0.137, *r* = 0.20, 95% CI -0.1 to 0.5). Unlike the results-guarantee item, this item’s baseline score was not close to the ceiling (3.12).

Subgroup analysis found no statistically significant differences in the pre-post change (delta) by training stage (Kruskal-Wallis, H(2) = 2.30, *P* = 0.32, eta-squared = 0.01), gender (Mann-Whitney *U* = 924.0, *P* = 0.19, *r* = -0.13), daily screen time (Kruskal-Wallis, H(3) = 1.74, *P* = 0.63, eta-squared = 0.008), or prior use of social media for medical purposes (Mann-Whitney *U* = 1209.5, *P* = 0.61, *r* = 0.05).

## Discussion

These findings reproduce, in a Brazilian sample, the paradox already described in the international literature: near-universal, high-frequency social media use among medical students coexists with low confidence in applying the ethical rules that govern that use [1,6,7]. The fact that 72 (71.3%) of students had heard of CFM social media rules, yet only 10 (9.9%) felt able to apply them safely, indicates that awareness of a norm’s existence is not equivalent to the ethical literacy needed to apply it - a distinction consistent with prior reports of cognitive dissonance among undergraduates regarding online professionalism [6,7].

The largest corrections were observed precisely in the scenarios most associated with the *hidden curriculum *described by Mostaghimi et al. and Souza et al. - informal therapeutic guidance via direct message, advertising sensationalism, and unrestricted exposure of surgical procedures [8,9]. These are conducts students are more likely to have witnessed being normalized by preceptors or peers rather than learned as formally prohibited; a single scenario-based workshop was enough to produce large, significant corrections in exactly these areas, which is consistent with the literature’s emphasis on active, case-based teaching as more effective than isolated theoretical lectures at correcting conduct absorbed informally [9,19].

The item on guaranteeing zero-error outcomes, whose baseline score (4.80) was already close to the scale’s ceiling, still showed a small but statistically significant gain (*P* = 0.026). This suggests that even where ethical recognition is already broadly established, the intervention contributed to reducing residual response variability, indicating that the instrument and the teaching strategy remained sensitive even under ceiling-effect conditions.

Two paradoxical and, in our view, related findings deserve treatment as a single phenomenon rather than isolated notes. The score for the scenario describing a recently permitted regulatory flexibility (a non-specialist physician advertising a non-titled subspecialty area, provided the lack of certification is disclosed) fell significantly after the workshop, as did the score for using social media as a valid channel for reliable communication during an epidemic - despite the latter describing a legitimate and already-recognized use of digital health communication. Taken together, these two findings suggest that concentrated exposure to the restrictions and sanctions of the Code of Medical Ethics may have produced an immediate conservative bias in students, who began to view with greater caution or suspicion not only the new resolution’s flexibilities but also already-established, legitimate uses of social media in health. This pattern is consistent with prior e-professionalism research indicating that a sudden increase in perceived regulation can inhibit conduct previously seen as harmless, including conduct that is, in fact, harmless. We propose the term *post-intervention overconservatism effect* for this pattern: concentrated exposure to the prohibitions of the Code of Medical Ethics appears to temporarily shift students’ judgment beyond what is warranted, penalizing legitimate conduct as well - a pedagogical side effect that future interventions should monitor and, ideally, correct by explicitly teaching examples of what is permitted, not only what is prohibited.

The single item that produced no detectable change - sharing de-identified clinical cases within a closed student group for learning purposes - is plausibly explained by the workshop not having dedicated specific time to that scenario, or by the scenario’s wording conflating a closed academic group with open public exposure, two situations that likely warrant different ethical treatment. This recommends both reinforcing this topic in future workshop editions and splitting the item into two distinct scenarios (closed group vs. open group) to reduce the ambiguity identified.

The absence of significant subgroup differences in knowledge gain by training stage, gender, screen time, or prior medical use of social media is itself a relevant finding. Unlike most educational-intervention studies in digital ethics, which frequently report gains concentrated among more advanced students or those with greater prior exposure to the topic, this result indicates that the workshop can be inserted at any point in the curriculum - from the basic cycle to internship - without loss of effectiveness, which considerably broadens its curricular applicability. It also implies that being further along in clinical training (and thus more exposed to real patient contact) does not, by itself, protect students from the boundary-blurring described elsewhere as a consequence of internship familiarity [10,11]; explicit teaching, not clinical exposure alone, appears to be the operative factor.

This study has several limitations. First, the absence of a control group limits the ability to establish definitive causality. Second, there was a substantial 22 (20%) loss to follow-up and a predominantly female sample, which may affect the generalizability of the outcomes across different demographics. Furthermore, the reliance on self-reported responses to hypothetical scenarios introduces potential social-desirability bias, and the measurement instrument has limited external validation. Consequently, the results support the conclusion that the intervention improved short-term ethical adequacy scores on hypothetical cases, rather than demonstrating long-term retention or definitive real-world behavioral changes, which would require future longitudinal and controlled studies.

For practical applicability in medical training programs, the key takeaways for educators are clear. Given the unexpectedly high volume of daily social media use among trainees, faculties must implement early, scenario-based digital ethics training. Educators should focus on bridging the gap between theoretical ethics and practical online behavior, utilizing real-world case studies to teach safe navigation of professional boundaries and patient privacy.

Future research should pursue longitudinal designs following students from internship into clinical practice, to verify the consolidation of ethical literacy over the medium and long term, including whether the conservative bias identified in the flexibility and public-health items persists or dissipates. Multicenter designs and direct observational analysis of students’ public profiles, cross-referenced with the theoretical knowledge demonstrated in questionnaires, would also help clarify whether stated knowledge translates into actual online conduct.

## Conclusions

This study shows that despite near-universal social media use among medical students, most initially lack the ethical-legal literacy required to safely navigate the advertising and confidentiality rules set out in Resolution No. 2,336/2023 of the Brazilian Federal Council of Medicine. A brief, scenario-based educational workshop produced a statistically significant, immediate improvement in short-term ethical adequacy scores on hypothetical cases, with no significant difference in effect across training stage, gender, or screen-time subgroups. While these findings do not establish long-term behavioral change, they support scenario-based teaching as a practical, low-cost strategy applicable across a heterogeneous student body at any stage of training. Finally, the post-intervention overconservatism effect identified here indicates that such interventions should also explicitly reinforce which digital conduct remains permitted, and not only which is prohibited, when they are built primarily around a code’s restrictions.

## Appendices

Appendix A

**Table 3:** Data collection instrument: pre-intervention questionnaire (demographics and baseline assessment).

Question / Item	Response Options
I declare that I have read the ICF, agree to participate in the research, and am 18 years of age or older on the date of participation in this research.	Yes / No
Pairing code (first two letters of mother's name + day of birth, two digits)	Open text response
Date of birth	Open text response
Gender	Male / Female / Other
Current semester of medical school	1st to 12th semester
Do you have active profile(s) on social networks? (e.g., Instagram, Facebook, TikTok, LinkedIn)	Yes / No
On which social network(s) do you have an active profile?	Instagram / Facebook / TikTok / YouTube / X / LinkedIn / Other / I do not have social media
Do you already use your social media for medicine-related activities (e.g., health information dissemination, interacting with patients, professional marketing)?	Yes / No
On average, how much time per day do you spend using social media?	Less than 1 hour / 1 to 3 hours / 3 to 5 hours / More than 5 hours
Have you ever heard of the Federal Council of Medicine (CFM) rules related to the use of social media?	Yes / No
What is your current perception regarding the knowledge of ethical rules applicable to the use of social media by physicians?	I know the rules well, feel confident, and know how to apply them / I have some notion, but do not feel confident applying it / I do not know the rules
Problem-scenario 1: A student may post a photograph of a patient's wound (without showing the face) on their social media after obtaining the patient's verbal consent.	5-Point Likert Scale (1 = Strongly disagree to 5 = Strongly agree)
Problem-scenario 2: A group of medical students may create a social media page to share clinical cases from the ward, including photos of tests and anonymized medical records, for learning purposes.	5-Point Likert Scale (1 = Strongly disagree to 5 = Strongly agree)
Problem-scenario 3: A physician may publish a prescription guide on their social media claiming to guarantee a 0% error rate on on-call shifts.	5-Point Likert Scale (1 = Strongly disagree to 5 = Strongly agree)
Problem-scenario 4: Social media can be considered a valid channel for transmitting reliable medical information during an epidemic.	5-Point Likert Scale (1 = Strongly disagree to 5 = Strongly agree)
Problem-scenario 5: A student should maintain a profile used exclusively for medical content, separate from personal posts, when using social media.	5-Point Likert Scale (1 = Strongly disagree to 5 = Strongly agree)
Problem-scenario 6: A physician may advertise on their social media that their clinic is the "only clinic in the city" equipped with a given device, claiming better results compared to others.	5-Point Likert Scale (1 = Strongly disagree to 5 = Strongly agree)
Problem-scenario 7: A surgery resident may record and publish a step-by-step surgical video on social media showing the execution of a procedure for teaching purposes.	5-Point Likert Scale (1 = Strongly disagree to 5 = Strongly agree)
Problem-scenario 8: A general practitioner with a postgraduate degree in endocrinology and sports medicine may maintain a professional Instagram profile and share content on prevention, healthy habits, and endocrinology topics, provided they clearly state they are not a board-certified specialist and correctly report their credentials.	5-Point Likert Scale (1 = Strongly disagree to 5 = Strongly agree)
Problem-scenario 9: A medical student may publish a real clinical case from an on-call shift on their social media, including age, diagnosis, and clinical course, provided they do not mention the patient's name or personal data (de-identified).	5-Point Likert Scale (1 = Strongly disagree to 5 = Strongly agree)
Problem-scenario 10: A medical student may publish posts about prevention and treatment based on scientific articles using phrases such as "this is the best treatment" or "this is the definitive solution".	5-Point Likert Scale (1 = Strongly disagree to 5 = Strongly agree)
Problem-scenario 11: A physician may answer patient questions about adverse effects of a prescribed medication via Instagram direct message, providing detailed therapeutic guidance.	5-Point Likert Scale (1 = Strongly disagree to 5 = Strongly agree)
Problem-scenario 12: A medical student may share a photo of an empty operating room on social media, with no visible patients, showing the environment and the materials used.	5-Point Likert Scale (1 = Strongly disagree to 5 = Strongly agree)
Problem-scenario 13: A physician who goes viral on social media with medical humor content related to health can harm the professional image of medicine.	5-Point Likert Scale (1 = Strongly disagree to 5 = Strongly agree)
Attitude 1: During my undergraduate studies, I received specific and sufficient instructions regarding medical ethics in the use of social media.	5-Point Likert Scale (1 = Strongly disagree to 5 = Strongly agree)
Attitude 2: Undergraduate programs should provide instructions to students on medical ethics and social media use.	5-Point Likert Scale (1 = Strongly disagree to 5 = Strongly agree)
Attitude 3: Physicians should seek out their state Medical Council (CRM) to become informed about ethical recommendations on social media.	5-Point Likert Scale (1 = Strongly disagree to 5 = Strongly agree)

Appendix B

**Table 4:** Data collection instrument: post-intervention additional feedback. Note: The full post-intervention questionnaire included the ICF verification and the repetition of the 16 scenarios and attitude questions detailed in Appendix A, followed by the final feedback section detailed in this table.

Question / Item	Response Options
After the intervention class, I feel more prepared to conduct myself ethically on social media related to medical practice.	5-Point Likert Scale (1 = Strongly disagree to 5 = Strongly agree)
Leave your feedback, if you wish, regarding participation in the class and the research:	Open text response
